# Supplementary material for: Environmental and spatial drivers of taxonomic, functional, and phylogenetic characteristics of bat communities in human-modified landscapes
Source: PeerJ. 2016 Oct 13;4:e2551. doi: 10.7717/peerj.2551 (PMC5068362; doi:10.7717/peerj.2551)
Supplement: Table S1 [file peerj-04-2551-s001.pdf]

Summary of landscape characteristics in the Caribbean lowlands of Costa Rica.

Table S1. Mean, standard deviation (SD), and range of each of nine landscape characteristics that were quantified at each of three focal scales (i.e. circles with a 1 km, 3 km, or 5 km radius) for sites in the Caribbean lowlands of Costa Rica (for definitions, formulae, and units of landscape metrics, see Table S4).

	1 km scale			3 km scale			5 km scale		
	Mean	SD	Range	Mean	SD	Range	Mean	SD	Range
Composition									
Percent forest	57.94	30.39	9.78 - 100.00	49.41	21.70	10.05 - 88.55	48.63	14.69	22.49 - 70.96
Percent pasture	28.75	23.08	0.00 - 77.80	37.61	16.72	10.09 - 70.55	39.68	10.86	25.00 - 60.15
Mean forest patch size	81.95	100.19	2.59 - 313.65	68.25	75.33	4.47 - 278.17	45.31	25.64	7.66 - 96.07
Forest patch density	1.82	1.67	0.32 - 6.68	1.41	1.00	0.32 - 4.32	1.32	0.58	0.74 - 3.09
Simpson's diversity	0.37	0.17	0.00 - 0.60	0.50	0.11	0.21 - 0.69	0.55	0.07	0.43 - 0.70
Configuration									
Mean forest proximity	96.98	128.46	0.00 - 408.63	752.85	758.58	8.59 - 2630.11	1300.00	1120.79	71.91 - 3361.66
Mean forest nearest neighbor	117.16	82.31	0.00 - 336.51	116.13	19.86	90.58 - 159.94	110.42	10.77	96.30 - 136.28
Mean forest patch shape	1.88	0.44	1.11 - 2.87	1.98	0.19	1.67 - 2.35	1.99	0.09	1.91 - 2.20
Forest edge density	40.07	20.07	0.00 - 72.85	46.40	14.99	17.66 - 69.50	48.46	9.58	33.95 - 62.70

Description of functional attributes.

Table S2. Attributes that reflect functional niche axes that were used to estimate variation of bat assemblages from the Caribbean lowlands of Costa Rica. Mensural attributes were measured as described in sources (see Table S3).

Type of data	Functional niche axis	Attribute	Trait value
Categorical	Diet	Fruit or plant	0, 1
		Nectar or pollen	0, 1
		Invertebrates	0, 1
		Vertebrates	0, 1
		Blood	0, 1
	Foraging location	Canopy	0, 1
		Subcanopy	0, 1
		Understory	0, 1
	Foraging strategy	Gleaning	0, 1
		Hover	0, 1
		Pounce	0, 1
	Roost type	Foliage	0, 1
		Bark or roots	0, 1
		Tree hole or termite nest	0, 1
		Man-made structures	0, 1
		Culvert or under large rocks	0, 1
		Cave, tunnels, mines or sewers	0, 1
Mensural	Size	Mass	Mean value (g)
		Forearm length	Mean value (mm)
	Skull	Greatest length of skull	Mean value (mm)
		Condylobasal length	Mean value (mm)
		Length of maxillary toothrow	Mean value (mm)
		Breadth across upper molars	Mean value (mm)
		Width across post-orbital constriction	Mean value (mm)
		Breadth of braincase	Mean value (mm)
	Wing	Wing loading	Mean value (mm)
		Aspect ratio	Mean value (mm)

Sources of functional attribute data.

Table S3. Sources of information for functional attributes of bat species were compiled from the literature and supplemented with data obtained from field measurements from the Caribbean lowlands of northeastern Costa Rica.

Functional niche axis	Source
Diet	Fleming, T.H., Hooper, E.T. & Wilson, D.E. (1972) Three Central American bat communities: structure, reproductive cycles, and movement patterns. *Ecology* , **53**, 556–569.
	Giannini, N.P. & Kalko, E.K.V. (2004) Trophic structure in a large assemblage of phyllostomid bats in Panama. *Oikos* , **105**, 209–220.
	Heithaus, E.R., Fleming, T.H. & Opler, P.A. (1975) Foraging patterns and resource utilization in seven species of bats in a seasonal tropical forest. *Ecology* , **56**, 841–854.
	Kalka, M. & Kalko, E.K.V. (2006) Gleaning bats as underestimated predators of herbivorous insects: diet of *Micronycteris microtis* (Phyllostomidae) in Panama. *Journal of Tropical Ecology* , **22**, 1–10.
	Linares, O.J. (1998) *Mamiferos de Venezuela* . Sociedad Conservacionista Audubon de Venezuela, Caracas, Venezuela.
	Rodriguez-Herrera, B., Medellín, R.A. & Timm, R.M. (2007) *Neotropical tent-roosting bats: field guide* . INBio, Santo Domingo de Heredia, Costa Rica.
	Tschapka, M. (2005) Reproduction of the bat *Glossophaga commissarisi* (Phyllostomidae: Glossophaginae) in the Costa Rican rain forest during frugivorous and nectarivorous periods. *Biotropica* , **37**, 409–415.
	York, H.A. & Billings, S.A. (2009) Stable-isotope analysis of diets of short-tailed fruit bats (Chiroptera: Phyllostomidae: *Carollia*). *Journal of Mammalogy* , **90**, 1469–1477.
Foraging location	Bernard E. (2001) Vertical stratification of bat communities in primary forests of Central Amazon, Brazil. *Journal of Tropical Ecology* , **17**, 115–126.
	Kalko, E.K.V. & Handley, C.O. (2001) Neotropical bats in the canopy: diversity, community structure, and implications for conservation. *Plant Ecology* , **153**, 319–333.
	Linares, O.J. (1998) *Mamiferos de Venezuela* . Sociedad Conservacionista Audubon de Venezuela, Caracas, Venezuela.
	Mammalian Species <http://www.science.smith.edu/msi/msiaccounts.html>
	Rex, K., Michener, R., Kunz, T.H. & Voigt, C.C. (2011) Vertical stratification of Neotropical leaf-nosed bats (Chiroptera: Phyllostomidae) revealed by stable carbon isotopes. *Journal of Tropical Ecology* , **27**, 211–222.
	Voigt, C.C. (2010) Insights into strata use of forest animals using the 'canopy effect'. *Biotropica* , **42**, 634–637.
Foraging strategy	Eisenberg, J.F. & Redford, K.H. (1999) *Mammals of the Neotropics. The Central Neotropics: Ecuador, Peru, Bolivia, Brazil.* The University of Chicago Press, Chicago, Illinois, USA and London, UK.
	Mammalian Species <http://www.science.smith.edu/msi/msiaccounts.html>
	Rodriguez-Herrera, B., Medellín, R.A. & Timm, R.M. (2007) *Neotropical tent-roosting bats: field guide* . INBio, Santo Domingo de Heredia, Costa Rica
Roost type	Eisenberg, J.F. & Redford, K.H. (1999) *Mammals of the Neotropics. The Central Neotropics: Panama, Colombia, Venezuela, Guyana, Suriname, French Guiana.* The University of Chicago Press, Chicago, Illinois, USA and London, UK.
	Linares, O.J. (1998) *Mamiferos de Venezuela* . Sociedad Conservacionista Audubon de Venezuela, Caracas, Venezuela.
	Mammalian Species <http://www.science.smith.edu/msi/msiaccounts.html>
	Rodriguez-Herrera, B., Medellín, R.A. & Timm, R.M. (2007) *Neotropical tent-roosting bats: field guide* . INBio, Santo Domingo de Heredia, Costa Rica.
Size	Cisneros, L.M. Measurements from specimens from northeastern Costa Rica. (unpublished)
	Eisenberg, J.F. & Redford, K.H. (1999) *Mammals of the Neotropics. The Central Neotropics: Panama, Colombia, Venezuela, Guyana, Suriname, French Guiana.* The University of Chicago Press, Chicago, Illinois, USA and London, UK.
	Linares, O.J. (1998) *Mamiferos de Venezuela* . Sociedad Conservacionista Audubon de Venezuela, Caracas, Venezuela.
	Mammalian Species <http://www.science.smith.edu/msi/msiaccounts.html>
Skull	Baker, R.J., Solari, S. & Hoffman, F.G. (2002) A new Central American species from the *Carollia brevicauda* complex. *Occasional Papers Museum of Texas Tech University* , **217**, 1–12.
	Mammalian Species <http://www.science.smith.edu/msi/msiaccounts.html>
	Simmons, N.B. (1996) A new species of *Micronycteris* (Chiroptera: Phyllostomidae) from northeastern Brazil, with comments on phylogenetic relationships. *American Museum Novitates* , **3158**, 1–34.
	Swanepoel, P. & Genoways, H.H. (1979) Morphometrics. *Biology of bats of the New World family Phyllostomatidae, Part III.* , (eds R.J. Baker, J.K Jones, Jr. & D.C. Carter), pp. 13–106. Special Publications Museum of Texas Tech University, Lubbock, Texas, USA.
Wing	Meyer, C.F. (2007) *Effects of rainforest fragmentation on Neotropical bats: land-bridge islands as a model system* . PhD thesis, Ulm University, Ulm, Germany.
	Norberg, U.M. & Rayner, J.M.V. (1987) Ecological morphology and flight in bats (Mammalia; Chiroptera): wing adaptations, flight performance, foraging strategy and echolocation. *Philosophical Transactions of the Royal Society London B* , **316**, 335–427.

Description of landscape metrics.

Table S4. Indices that quantified landscape structure at each of three focal scales for each of the 15 sampling sites based on a 2011 land cover map.

Landscape index	Formula	Variables	Description
Composition			
Percent forest/pasture	$\frac{\sum_{j=1}^{n} a_{ij}}{A}(100)$	a_{ij}, area (m^2) of patch ij; A, total landscape area (m^2).	Percent of the total area of the focal scale occupied by a particular land cover type (i.e. forest or pasture).
Mean forest patch size	$\frac{\sum_{j=1}^{n} a_{ij}\left(\frac{1}{10,000}\right)}{n_i}$	a_{ij}, area (m^2) of patch ij; n_i, number of patches of land cover type i.	Average area of all forest patches (divided by 10,000 to convert to hectares) within a focal scale.
Forest patch density	$\frac{n_i}{A}(10,000)(100)$	n_i, number of patches of land cover type i; A, total landscape area (m^2).	Number of forest patches divided by total area of the focal scale (multiplied 10,000 and 100 to convert to 100 hectares).
Simpson's diversity	$1-\sum_{i=1}^{m} P_i^2$	P_i, proportion of landscape occupied by land cover type i.	Measures landscape heterogeneity by considering proportions of all land cover types within a focal scale.
Configuration			
Mean forest proximity	$\frac{\sum_{j}^{n}\sum_{s=1}^{n}\frac{a_{ijs}}{h_{ijs}^2}}{n_i}$	a_{ijs}, area (m^2) of patches ijs within focal scale; h_{ijs}, distance (m) between patch ijs and nearest neighbor patch ijs, based on patch edge-to-edge distance; n_i, number of patches type i.	Average of the sum of forest patch area divided by the edge-to-edge distance squared between the focal patch and the nearest patch for all forest patches within a focal scale.
Mean forest nearest neighbor	$\frac{\sum_{j=1}^{n} h_{ij}}{n_i}$	h_{ij}, distance (m) between patch ij and nearest neighbor patch of type i, based on patch edge-to-edge distance; n_i, number of patches type i.	Average minimum edge-to-edge distance between all possible pairwise patches of forest in a focal scale.
Mean forest patch shape	$\frac{\sum_{j=1}^{n}\frac{0.25\,p_{ij}}{\sqrt{a_{ij}}}}{n_i}$	p_{ij}, perimeter (m) of patch ij; a_{ij}, area (m^2) of patch ij; n_i, number of patches of land cover type i.	Average of forest patch perimeter divided by square root of patch area, adjusted by a constant to adjust for a square standard, within a focal scale.
Forest edge density	$\frac{\sum_{k=1}^{m} e_{ik}}{A}(10,000)$	e_{ik}, total length (m) of edge of all patches type i; A, total landscape area (m^2).	Total length of edge of forest patches divided by total area of the focal scale (multiplied by 10,000 to convert to hectares).

Proportion of functional or phylogenetic variation due to composition or due to dispersion.

Table S5. Proportion of the total functional or phylogenetic variation (sum-of-squares) that was due to the composition component or the dispersion component for each of the eight functional approaches and for the phylogenetic approach.

	Composition	Dispersion
Dry season		
Functional — all	0.10	0.90
Functional — diet	0.03	0.97
Functional — foraging location	0.14	0.86
Functional — foraging strategy	0.15	0.85
Functional — roost	0.03	0.97
Functional — size	0.09	0.91
Functional — skull	0.08	0.92
Functional — wing	0.09	0.91
Phylogenetic	0.06	0.94
Wet season		
Functional — all	0.07	0.93
Functional — diet	0.04	0.96
Functional — foraging location	0.07	0.93
Functional — foraging strategy	0.08	0.92
Functional — roost	0.02	0.98
Functional — size	0.08	0.92
Functional — skull	0.08	0.92
Functional — wing	0.07	0.93
Phylogenetic	0.04	0.96

Results from three weighted least-square regressions using environmental and spatial predictors for species composition, functional dispersion or phylogenetic dispersion.

Table S6. For each combination of season and scale, adjusted R^2 of the model with both sets of predictor variables [abc], the model with environmental variables [ab], and the model with the spatial variable [bc] for (1) taxonomic structure based on species composition, (2) functional dispersion based on all attributes, and (3) phylogenetic dispersion. Negative values of adjusted R^2 should be interpreted as zeros (Legrendre 2008).

	1 km scale			3 km scale			5 km scale		
	[abc]	[ab]	[bc]	[abc]	[ab]	[bc]	[abc]	[ab]	[bc]
Dry season									
Taxonomic	0.15	0.17	0.04	0.31	0.25	0.04	0.39	0.44	0.04
Functional — all	1.00	1.00	0.94	0.99	0.99	0.94	1.00	1.00	0.94
Phylogenetic	1.00	0.99	0.97	0.98	0.98	0.97	0.99	0.99	0.97
Wet season									
Taxonomic	-0.02	0.03	0.07	0.15	-0.02	0.07	0.13	0.17	0.07
Functional — all	0.98	0.98	0.95	0.99	0.99	0.95	0.99	0.99	0.95
Phylogenetic	0.99	0.99	0.97	1.00	1.00	0.97	1.00	1.00	0.97

Results from variation partitioning for functional dispersion based on different niche axes.

Table S7. For each combination of season and scale, adjusted R^2 of the model with both sets of predictor variables [abc], the model with environmental variables [ab], the model with the spatial variable [bc] and the four fractions ([a], [b], [c], and [d]) for functional dispersion (s_F) based on different functional niche axes. Fractions [a] and [c] are the unique contributions of environment and space, respectively. Fraction [b] is the shared contributions of environmental and spatial predictors, and fraction [d] is the residual variation. Testable model fractions (i.e. unique contributions) that were significant are indicated by superscript symbols ($^@$, $0.10 \geq P > 0.05$; *, $P \leq 0.05$).

	1 km scale						
	[abc]	[ab]	[bc]	[a]	[b]	[c]	[d]
Dry season							
Diet	0.94	0.90	0.75	0.19	0.71	0.04$^@$	0.06
Foraging location	0.99	0.98	0.84	0.15	0.83	0.01	0.01
Foraging strategy	0.91	0.89	0.50	0.41	0.49	0.01	0.09
Roost	0.97	0.97	0.90	0.07	0.90	< 0.01	0.03
Size	0.98	0.97	0.91	0.07	0.90	0.01	0.02
Skull	0.97	0.96	0.89	0.08	0.88	0.01	0.03
Wing	0.99	0.99	0.97	0.03	0.96	< 0.01	0.01
Wet season							
Diet	0.99	0.99	0.95	0.04	0.95	< 0.01	0.01
Foraging location	0.99	0.99	0.96	0.03	0.96	< 0.01	0.01
Foraging strategy	0.95	0.95	0.52	0.43	0.52	< 0.01	0.05
Roost	0.99	0.99	0.91	0.09	0.91	< 0.01	0.01
Size	0.93	0.93	0.75	0.18	0.75	< 0.01	0.07
Skull	0.93	0.93	0.77	0.17	0.76	< 0.01	0.07
Wing	0.97	0.97	0.88	0.09	0.88	< 0.01	0.03
	3 km scale						
	[abc]	[ab]	[bc]	[a]	[b]	[c]	[d]
Dry season							
Diet	0.93	0.88	0.75	0.18	0.70	0.05*	0.07
Foraging location	0.97	0.97	0.84	0.13	0.84	< 0.01	0.03
Foraging strategy	0.91	0.88	0.50	0.41	0.47	0.03	0.09
Roost	0.99	0.99	0.90	0.09	0.90	< 0.01	0.01
Size	0.94	0.94	0.91	0.03	0.90	0.01	0.06
Skull	0.93	0.92	0.89	0.04	0.88	0.01	0.07
Wing	0.98	0.98	0.97	0.01	0.96	< 0.01	0.02
Wet season							
Diet	1.00	1.00	0.95	0.05$^@$	0.95	< 0.01	< 0.01
Foraging location	0.99	0.99	0.96	0.03	0.96	< 0.01	0.01
Foraging strategy	0.96	0.96	0.52	0.44	0.51	0.01	0.04
Roost	0.99	0.99	0.91	0.09	0.91	< 0.01	0.01
Size	0.92	0.88	0.75	0.17	0.71	0.04	0.08
Skull	0.93	0.89	0.77	0.17	0.73	0.04	0.07
Wing	0.97	0.96	0.88	0.09	0.87	0.01	0.03
	5 km scale						
	[abc]	[ab]	[bc]	[a]	[b]	[c]	[d]
Dry season							
Diet	0.91	0.91	0.75	0.16	0.75	< 0.01	0.09
Foraging location	0.99	0.99	0.84	0.15	0.84	< 0.01	0.01
Foraging strategy	0.98	0.96	0.50	0.48	0.48	0.02	0.02
Roost	1.00	0.99	0.90	0.10	0.90	< 0.01	< 0.01
Size	0.98	0.95	0.91	0.07	0.88	0.03*	0.02
Skull	0.97	0.94	0.89	0.09	0.85	0.03*	0.03
Wing	0.99	0.98	0.97	0.03	0.95	0.01*	0.01
Wet season							
Diet	0.99	0.99	0.95	0.04	0.95	< 0.01	0.01
Foraging location	0.99	0.99	0.96	0.03	0.96	< 0.01	0.01
Foraging strategy	0.99	0.95	0.52	0.47	0.48	0.04	0.01
Roost	0.99	0.99	0.91	0.08	0.91	< 0.01	0.01
Size	0.93	0.90	0.75	0.18	0.72	0.03	0.07
Skull	0.94	0.91	0.77	0.17	0.74	0.03	0.06
Wing	0.97	0.96	0.88	0.09	0.87	0.01	0.03

Results from variation partitioning for functional or phylogenetic composition.

Table S8. For each combination of season and scale, adjusted percentages of unique contributions of environmental [a] and spatial [c] predictors for functional or phylogenetic composition. Fraction [b] is the shared contribution of environmental and spatial predictors, and fraction [d] is the residual variation. Negative values of [b] can occur when explanatory variables are correlated but have strong and opposite effects on the response variable, or when explanatory variables have a weak correlation with the response variable but strong correlation with other explanatory variables that are correlated with the response variable (Peres-Neto et al. 2006). Testable model fractions (i.e. unique contributions) that were significant are indicated by superscript symbols ($^{@}$, $0.10 \geq P > 0.05$; *, $P \leq 0.05$).

	1 km scale				3 km scale				5 km scale			
	[a]	[b]	[c]	[d]	[a]	[b]	[c]	[d]	[a]	[b]	[c]	[d]
Dry season												
Functional — all	1.85	-1.09	< 0.01	0.24	2.02	-1.44	0.35	0.07	2.01	-1.09	0.01	0.07
Functional — diet	1.64	-1.43	0.23	0.56	1.90	-1.29	0.09	0.30	1.41	-1.21	0.01	0.79
Functional — foraging location	2.24	-1.47	0.08	0.16	2.20	-1.52	0.12	0.20	2.22	-1.43	0.03	0.18
Functional — foraging strategy	2.59	-2.22	0.20	0.43	2.05	-2.04	0.02	0.97	2.59	-2.34	0.32	0.44
Functional — roost	1.91	-1.74	< 0.01	0.83	2.12	-2.01	0.26	0.62	2.44	-2.34	0.60	0.30
Functional — size	2.52	-2.52	0.33	0.67	1.18	-2.56	0.38	2.00	2.50	-2.60	0.42	0.68
Functional — skull	2.34	-2.34	0.27	0.73	1.04	-2.43	0.37	2.03	2.42	-2.49	0.42	0.65
Functional — wing	2.92	-2.66	0.28	0.46	1.77	-2.53	0.15	1.61	2.80	-2.59	0.21	0.59
Phylogenetic	2.49	-2.10	0.01	0.60	2.52	-2.32	0.24	0.57	2.68	-2.37	0.28	0.41
Wet season												
Functional — all	2.28	-1.63	0.02	0.33	1.76	-2.54	0.93*	0.85	2.23	-1.76	0.15	0.38
Functional — diet	1.83	-1.30	< 0.01	0.47	1.76	-2.19	0.89$^{@}$	0.55	2.00	-1.35	0.05	0.30
Functional — foraging location	2.29	-1.48	0.01	0.18	1.78	-2.12	0.64$^{@}$	0.69	2.23	-1.50	0.03	0.24
Functional — foraging strategy	2.87	-2.23	0.07	0.29	2.68	-2.16	< 0.01	0.48	2.97	-2.58	0.41	0.19
Functional — roost	1.75	-2.17	< 0.01	1.41	2.58	-2.92	0.75	0.59	2.45	-2.17	0.01	0.71
Functional — size	2.68	-2.22	0.04	0.50	1.97	-2.88	0.69*	1.21	2.75	-2.52	0.33	0.44
Functional — skull	2.62	-2.11	0.03	0.46	1.96	-2.81	0.73*	1.12	2.67	-2.43	0.35	0.41
Functional — wing	2.53	-2.05	0.01	0.51	2.16	-2.62	0.59$^{@}$	0.88	2.82	-2.48	0.45$^{@}$	0.21
Phylogenetic	2.60	-2.02	0.01	0.42	2.27	-2.31	0.29	0.74	2.82	-2.36	0.35	0.19

Spatial autocorrelation of functional or phylogenetic dispersion.

Table S9. Spatial autocorrelation of functional dispersion (based on all niche axes and individual niche axes) and phylogenetic dispersion of bat communities in the Caribbean lowlands of Costa Rica for the dry and wet seasons. Significance ($p \leq 0.05$) indicates spatial autocorrelation.

	Observed Moran's I	Expected Moran's I	SD	p
Dry season				
Functional — all	-0.02	-0.07	0.06	0.38
Functional — diet	-0.13	-0.07	0.06	0.29
Functional — foraging location	-0.03	-0.07	0.05	0.39
Functional — foraging strategy	-0.02	-0.07	0.06	0.34
Functional — roost	-0.09	-0.07	0.06	0.73
Functional — size	-0.11	-0.07	0.06	0.54
Functional — skull	-0.10	-0.07	0.06	0.56
Functional — wing	-0.09	-0.07	0.05	0.72
Phylogenetic	-0.08	-0.07	0.05	0.82
Wet season				
Functional — all	-0.08	-0.07	0.05	0.80
Functional — diet	-0.06	-0.07	0.06	0.88
Functional — foraging location	-0.05	-0.07	0.05	0.65
Functional — foraging strategy	0.00	-0.07	0.05	0.20
Functional — roost	-0.09	-0.07	0.06	0.80
Functional — size	-0.10	-0.07	0.05	0.63
Functional — skull	-0.09	-0.07	0.05	0.75
Functional — wing	-0.08	-0.07	0.05	0.92
Phylogenetic	-0.13	-0.07	0.06	0.25

Spatial autocorrelation of landscape characteristics at each of three focal scales.

Table S10. Spatial autocorrelation of landscape characteristics at each of three focal scales (i.e. 1, 3, and 5 km radius) in the Caribbean lowlands of Costa Rica. Significance ($p \leq 0.05$) indicates spatial autocorrelation.

	1 km scale				3 km scale				5 km scale			
	Observed Moran's I	Expected Moran's I	SD	p	Observed Moran's I	Expected Moran's I	SD	p	Observed Moran's I	Expected Moran's I	SD	p
Percent forest	-0.04	-0.07	0.06	0.54	-0.06	-0.07	0.06	0.89	-0.05	-0.07	0.06	0.64
Percent pasture	-0.07	-0.07	0.06	0.93	-0.06	-0.07	0.06	0.78	0.02	-0.07	0.06	0.12
Mean forest patch size	-0.12	-0.07	0.05	0.37	-0.11	-0.07	0.05	0.38	-0.11	-0.07	0.06	0.50
Forest patch density	-0.09	-0.07	0.05	0.64	-0.09	-0.07	0.05	0.74	-0.08	-0.07	0.04	0.80
Simpson's diversity	-0.14	-0.07	0.05	0.24	-0.13	-0.07	0.05	0.26	-0.04	-0.07	0.05	0.56
Mean forest proximity	-0.08	-0.07	0.05	0.84	-0.05	-0.07	0.05	0.72	0.03	-0.07	0.06	0.06
Mean forest nearest neighbor	-0.04	-0.07	0.05	0.52	-0.02	-0.07	0.05	0.33	-0.03	-0.07	0.05	0.42
Mean forest patch shape	-0.09	-0.07	0.05	0.77	-0.06	-0.07	0.06	0.85	0.01	-0.07	0.05	0.15
Forest edge density	-0.05	-0.07	0.06	0.75	-0.04	-0.07	0.06	0.57	-0.02	-0.07	0.06	0.34
