# Supplementary material for: Environmental and spatial drivers of taxonomic, functional, and phylogenetic characteristics of bat communities in human-modified landscapes
Source: PeerJ. 2016 Oct 13;4:e2551. doi: 10.7717/peerj.2551 (PMC5068362; doi:10.7717/peerj.2551)
Supplement: Table S2 [file peerj-04-2551-s002.pdf]

Description of functional attributes.

Table S2. Attributes that reflect functional niche axes that were used to estimate variation of bat assemblages from the Caribbean lowlands of Costa Rica. Mensural attributes were measured as described in sources (see Table S3).

Type of data	Functional niche axis	Attribute	Trait value
Categorical	Diet	Fruit or plant	0, 1
		Nectar or pollen	0, 1
		Invertebrates	0, 1
		Vertebrates	0, 1
		Blood	0, 1
	Foraging location	Canopy	0, 1
		Subcanopy	0, 1
		Understory	0, 1
	Foraging strategy	Gleaning	0, 1
		Hover	0, 1
		Pounce	0, 1
	Roost type	Foliage	0, 1
		Bark or roots	0, 1
		Tree hole or termite nest	0, 1
		Man-made structures	0, 1
		Culvert or under large rocks	0, 1
		Cave, tunnels, mines or sewers	0, 1
Mensural	Size	Mass	Mean value (g)
		Forearm length	Mean value (mm)
	Skull	Greatest length of skull	Mean value (mm)
		Condylobasal length	Mean value (mm)
		Length of maxillary toothrow	Mean value (mm)
		Breadth across upper molars	Mean value (mm)
		Width across post-orbital constriction	Mean value (mm)
		Breadth of braincase	Mean value (mm)
	Wing	Wing loading	Mean value (mm)
		Aspect ratio	Mean value (mm)
