# Supplementary material for: Environmental and spatial drivers of taxonomic, functional, and phylogenetic characteristics of bat communities in human-modified landscapes
Source: PeerJ. 2016 Oct 13;4:e2551. doi: 10.7717/peerj.2551 (PMC5068362; doi:10.7717/peerj.2551)
Supplement: Table S3 [file peerj-04-2551-s003.pdf]

Sources of functional attribute data.

Table S3. Sources of information for functional attributes of bat species were compiled from the literature and supplemented with data obtained from field measurements from the Caribbean lowlands of northeastern Costa Rica.

Functional niche axis	Source
Diet	Fleming, T.H., Hooper, E.T. & Wilson, D.E. (1972) Three Central American bat communities: structure, reproductive cycles, and movement patterns. *Ecology*, **53**, 556–569.<br>Giannini, N.P. & Kalko, E.K.V. (2004) Trophic structure in a large assemblage of phyllostomid bats in Panama. *Oikos*, **105**, 209–220.<br>Heithaus, E.R., Fleming, T.H. & Opler, P.A. (1975) Foraging patterns and resource utilization in seven species of bats in a seasonal tropical forest. *Ecology*, **56**, 841–854.<br>Kalka, M. & Kalko, E.K.V. (2006) Gleaning bats as underestimated predators of herbivorous insects: diet of *Micronycteris microtis* (Phyllostomidae) in Panama. *Journal of Tropical Ecology*, **22**, 1–10.<br>Linares, O.J. (1998) *Mamiferos de Venezuela*. Sociedad Conservacionista Audubon de Venezuela, Caracas, Venezuela.<br>Rodriguez-Herrera, B., Medellín, R.A. & Timm, R.M. (2007) *Neotropical tent-roosting bats: field guide*. INBio, Santo Domingo de Heredia, Costa Rica.<br>Tschapka, M. (2005) Reproduction of the bat *Glossophaga commissarisi* (Phyllostomidae: Glossophaginae) in the Costa Rican rain forest during frugivorous and nectarivorous periods. *Biotropica*, **37**, 409–415.<br>York, H.A. & Billings, S.A. (2009) Stable-isotope analysis of diets of short-tailed fruit bats (Chiroptera: Phyllostomidae: *Carollia*). *Journal of Mammalogy*, **90**, 1469–1477.
Foraging location	Bernard E. (2001) Vertical stratification of bat communities in primary forests of Central Amazon, Brazil. *Journal of Tropical Ecology*, **17**, 115–126.<br>Kalko, E.K.V. & Handley, C.O. (2001) Neotropical bats in the canopy: diversity, community structure, and implications for conservation. *Plant Ecology*, **153**, 319–333.<br>Linares, O.J. (1998) *Mamiferos de Venezuela*. Sociedad Conservacionista Audubon de Venezuela, Caracas, Venezuela.<br>*Mammalian Species* <http://www.science.smith.edu/msi/msiaccounts.html><br>Rex, K., Michener, R., Kunz, T.H. & Voigt, C.C. (2011) Vertical stratification of Neotropical leaf-nosed bats (Chiroptera: Phyllostomidae) revealed by stable carbon isotopes. *Journal of Tropical Ecology*, **27**, 211–222.<br>Voigt, C.C. (2010) Insights into strata use of forest animals using the 'canopy effect'. *Biotropica*, **42**, 634–637.
Foraging strategy	Eisenberg, J.F. & Redford, K.H. (1999) *Mammals of the Neotropics. The Central Neotropics: Ecuador, Peru, Bolivia, Brazil.* The University of Chicago Press, Chicago, Illinois, USA and London, UK.<br>*Mammalian Species* <http://www.science.smith.edu/msi/msiaccounts.html><br>Rodriguez-Herrera, B., Medellín, R.A. & Timm, R.M. (2007) *Neotropical tent-roosting bats: field guide*. INBio, Santo Domingo de Heredia, Costa Rica
Roost type	Eisenberg, J.F. & Redford, K.H. (1999) *Mammals of the Neotropics. The Central Neotropics: Panama, Colombia, Venezuela, Guyana, Suriname, French Guiana.* The University of Chicago Press, Chicago, Illinois, USA and London, UK.<br>Linares, O.J. (1998) *Mamiferos de Venezuela*. Sociedad Conservacionista Audubon de Venezuela, Caracas, Venezuela.<br>*Mammalian Species* <http://www.science.smith.edu/msi/msiaccounts.html><br>Rodriguez-Herrera, B., Medellín, R.A. & Timm, R.M. (2007) *Neotropical tent-roosting bats: field guide*. INBio, Santo Domingo de Heredia, Costa Rica.
Size	Cisneros, L.M. Measurements from specimens from northeastern Costa Rica. (unpublished)<br>Eisenberg, J.F. & Redford, K.H. (1999) *Mammals of the Neotropics. The Central Neotropics: Panama, Colombia, Venezuela, Guyana, Suriname, French Guiana.* The University of Chicago Press, Chicago, Illinois, USA and London, UK.<br>Linares, O.J. (1998) *Mamiferos de Venezuela*. Sociedad Conservacionista Audubon de Venezuela, Caracas, Venezuela.<br>*Mammalian Species* <http://www.science.smith.edu/msi/msiaccounts.html>
Skull	Baker, R.J., Solari, S. & Hoffman, F.G. (2002) A new Central American species from the *Carollia brevicauda* complex. *Occasional Papers Museum of Texas Tech University*, **217**, 1–12.<br>*Mammalian Species* <http://www.science.smith.edu/msi/msiaccounts.html><br>Simmons, N.B. (1996) A new species of *Micronycteris* (Chiroptera: Phyllostomidae) from northeastern Brazil, with comments on phylogenetic relationships. *American Museum Novitates*, **3158**, 1–34.<br>Swanepoel, P. & Genoways, H.H. (1979) Morphometrics. *Biology of bats of the New World family Phyllostomatidae, Part III.*, (eds R.J. Baker, J.K Jones, Jr. & D.C. Carter), pp. 13–106. Special Publications Museum of Texas Tech University, Lubbock, Texas, USA.
Wing	Meyer, C.F. (2007) *Effects of rainforest fragmentation on Neotropical bats: land-bridge islands as a model system*. PhD thesis, Ulm University, Ulm, Germany.<br>Norberg, U.M. & Rayner, J.M.V. (1987) Ecological morphology and flight in bats (Mammalia; Chiroptera): wing adaptations, flight performance, foraging strategy and echolocation. *Philosophical Transactions of the Royal Society London B*, **316**, 335–427.
