# Supplementary material for: Environmental and spatial drivers of taxonomic, functional, and phylogenetic characteristics of bat communities in human-modified landscapes
Source: PeerJ. 2016 Oct 13;4:e2551. doi: 10.7717/peerj.2551 (PMC5068362; doi:10.7717/peerj.2551)
Supplement: Table S4 [file peerj-04-2551-s004.pdf]

Description of landscape metrics.

Table S4. Indices that quantified landscape structure at each of three focal scales for each of the 15 sampling sites based on a 2011 land cover map.

Landscape index	Formula	Variables	Description
Composition			
Percent forest/pasture	$\frac{\sum_{j=1}^{n} a_{ij}}{A}(100)$	a_{ij}, area (m^2) of patch ij; A, total landscape area (m^2).	Percent of the total area of the focal scale occupied by a particular land cover type (i.e. forest or pasture).
Mean forest patch size	$\frac{\sum_{j=1}^{n} a_{ij}\left(\frac{1}{10{,}000}\right)}{n_i}$	a_{ij}, area (m^2) of patch ij; n_i, number of patches of land cover type i.	Average area of all forest patches (divided by 10,000 to convert to hectares) within a focal scale.
Forest patch density	$\frac{n_i}{A}(10{,}000)(100)$	n_i, number of patches of land cover type i; A, total landscape area (m^2).	Number of forest patches divided by total area of the focal scale (multiplied 10,000 and 100 to convert to 100 hectares).
Simpson's diversity	$1-\sum_{i=1}^{m} P_i^2$	P_i, proportion of landscape occupied by land cover type i.	Measures landscape heterogeneity by considering proportions of all land cover types within a focal scale.
Configuration			
Mean forest proximity	$\frac{\sum_{j}^{n}\sum_{s=1}^{n}\frac{a_{ijs}}{h_{ijs}^2}}{n_i}$	a_{ijs}, area (m^2) of patches ijs within focal scale; h_{ijs}, distance (m) between patch ijs and nearest neighbor patch ijs, based on patch edge-to-edge distance; n_i, number of patches type i.	Average of the sum of forest patch area divided by the edge-to-edge distance squared between the focal patch and the nearest patch for all forest patches within a focal scale.
Mean forest nearest neighbor	$\frac{\sum_{j=1}^{n} h_{ij}}{n_i}$	h_{ij}, distance (m) between patch ij and nearest neighbor patch of type i, based on patch edge-to-edge distance; n_i, number of patches type i.	Average minimum edge-to-edge distance between all possible pairwise patches of forest in a focal scale.
Mean forest patch shape	$\frac{\sum_{j=1}^{n}\frac{0.25\,p_{ij}}{\sqrt{a_{ij}}}}{n_i}$	p_{ij}, perimeter (m) of patch ij; a_{ij}, area (m^2) of patch ij; n_i, number of patches of land cover type i.	Average of forest patch perimeter divided by square root of patch area, adjusted by a constant to adjust for a square standard, within a focal scale.
Forest edge density	$\frac{\sum_{k=1}^{m} e_{ik}}{A}(10{,}000)$	e_{ik}, total length (m) of edge of all patches type i; A, total landscape area (m^2).	Total length of edge of forest patches divided by total area of the focal scale (multiplied by 10,000 to convert to hectares).
