# Supplementary material for: Environmental and spatial drivers of taxonomic, functional, and phylogenetic characteristics of bat communities in human-modified landscapes
Source: PeerJ. 2016 Oct 13;4:e2551. doi: 10.7717/peerj.2551 (PMC5068362; doi:10.7717/peerj.2551)
Supplement: Table S5 [file peerj-04-2551-s005.pdf]

Proportion of functional or phylogenetic variation due to composition or due to dispersion.

Table S5. Proportion of the total functional or phylogenetic variation (sum-of-squares) that was due to the composition component or the dispersion component for each of the eight functional approaches and for the phylogenetic approach.

	Composition	Dispersion
Dry season		
Functional — all	0.10	0.90
Functional — diet	0.03	0.97
Functional — foraging location	0.14	0.86
Functional — foraging strategy	0.15	0.85
Functional — roost	0.03	0.97
Functional — size	0.09	0.91
Functional — skull	0.08	0.92
Functional — wing	0.09	0.91
Phylogenetic	0.06	0.94
Wet season		
Functional — all	0.07	0.93
Functional — diet	0.04	0.96
Functional — foraging location	0.07	0.93
Functional — foraging strategy	0.08	0.92
Functional — roost	0.02	0.98
Functional — size	0.08	0.92
Functional — skull	0.08	0.92
Functional — wing	0.07	0.93
Phylogenetic	0.04	0.96
