# Supplementary material for: Environmental and spatial drivers of taxonomic, functional, and phylogenetic characteristics of bat communities in human-modified landscapes
Source: PeerJ. 2016 Oct 13;4:e2551. doi: 10.7717/peerj.2551 (PMC5068362; doi:10.7717/peerj.2551)
Supplement: Table S6 [file peerj-04-2551-s006.pdf]

Results from three weighted least-square regressions using environmental and spatial predictors for species composition, functional dispersion or phylogenetic dispersion.

Table S6. For each combination of season and scale, adjusted R^2 of the model with both sets of predictor variables [abc], the model with environmental variables [ab], and the model with the spatial variable [bc] for (1) taxonomic structure based on species composition, (2) functional dispersion based on all attributes, and (3) phylogenetic dispersion. Negative values of adjusted R^2 should be interpreted as zeros (Legrendre 2008).

	1 km scale			3 km scale			5 km scale		
	[abc]	[ab]	[bc]	[abc]	[ab]	[bc]	[abc]	[ab]	[bc]
Dry season									
Taxonomic	0.15	0.17	0.04	0.31	0.25	0.04	0.39	0.44	0.04
Functional — all	1.00	1.00	0.94	0.99	0.99	0.94	1.00	1.00	0.94
Phylogenetic	1.00	0.99	0.97	0.98	0.98	0.97	0.99	0.99	0.97
Wet season									
Taxonomic	-0.02	0.03	0.07	0.15	-0.02	0.07	0.13	0.17	0.07
Functional — all	0.98	0.98	0.95	0.99	0.99	0.95	0.99	0.99	0.95
Phylogenetic	0.99	0.99	0.97	1.00	1.00	0.97	1.00	1.00	0.97
