# Supplementary material for: Environmental and spatial drivers of taxonomic, functional, and phylogenetic characteristics of bat communities in human-modified landscapes
Source: PeerJ. 2016 Oct 13;4:e2551. doi: 10.7717/peerj.2551 (PMC5068362; doi:10.7717/peerj.2551)
Supplement: Table S7 [file peerj-04-2551-s007.pdf]

Results from variation partitioning for functional dispersion based on different niche axes.

Table S7. For each combination of season and scale, adjusted R^2 of the model with both sets of predictor variables [abc], the model with environmental variables [ab], the model with the spatial variable [bc] and the four fractions ([a], [b], [c], and [d]) for functional dispersion (s_F) based on different functional niche axes. Fractions [a] and [c] are the unique contributions of environment and space, respectively. Fraction [b] is the shared contributions of environmental and spatial predictors, and fraction [d] is the residual variation. Testable model fractions (i.e. unique contributions) that were significant are indicated by superscript symbols ($^@$, $0.10 \geq P > 0.05$; *, $P \leq 0.05$).

	1 km scale						
	[abc]	[ab]	[bc]	[a]	[b]	[c]	[d]
Dry season							
Diet	0.94	0.90	0.75	0.19	0.71	0.04$^@$	0.06
Foraging location	0.99	0.98	0.84	0.15	0.83	0.01	0.01
Foraging strategy	0.91	0.89	0.50	0.41	0.49	0.01	0.09
Roost	0.97	0.97	0.90	0.07	0.90	< 0.01	0.03
Size	0.98	0.97	0.91	0.07	0.90	0.01	0.02
Skull	0.97	0.96	0.89	0.08	0.88	0.01	0.03
Wing	0.99	0.99	0.97	0.03	0.96	< 0.01	0.01
Wet season							
Diet	0.99	0.99	0.95	0.04	0.95	< 0.01	0.01
Foraging location	0.99	0.99	0.96	0.03	0.96	< 0.01	0.01
Foraging strategy	0.95	0.95	0.52	0.43	0.52	< 0.01	0.05
Roost	0.99	0.99	0.91	0.09	0.91	< 0.01	0.01
Size	0.93	0.93	0.75	0.18	0.75	< 0.01	0.07
Skull	0.93	0.93	0.77	0.17	0.76	< 0.01	0.07
Wing	0.97	0.97	0.88	0.09	0.88	< 0.01	0.03
	3 km scale						
	[abc]	[ab]	[bc]	[a]	[b]	[c]	[d]
Dry season							
Diet	0.93	0.88	0.75	0.18	0.70	0.05*	0.07
Foraging location	0.97	0.97	0.84	0.13	0.84	< 0.01	0.03
Foraging strategy	0.91	0.88	0.50	0.41	0.47	0.03	0.09
Roost	0.99	0.99	0.90	0.09	0.90	< 0.01	0.01
Size	0.94	0.94	0.91	0.03	0.90	0.01	0.06
Skull	0.93	0.92	0.89	0.04	0.88	0.01	0.07
Wing	0.98	0.98	0.97	0.01	0.96	< 0.01	0.02
Wet season							
Diet	1.00	1.00	0.95	0.05$^@$	0.95	< 0.01	< 0.01
Foraging location	0.99	0.99	0.96	0.03	0.96	< 0.01	0.01
Foraging strategy	0.96	0.96	0.52	0.44	0.51	0.01	0.04
Roost	0.99	0.99	0.91	0.09	0.91	< 0.01	0.01
Size	0.92	0.88	0.75	0.17	0.71	0.04	0.08
Skull	0.93	0.89	0.77	0.17	0.73	0.04	0.07
Wing	0.97	0.96	0.88	0.09	0.87	0.01	0.03
	5 km scale						
	[abc]	[ab]	[bc]	[a]	[b]	[c]	[d]
Dry season							
Diet	0.91	0.91	0.75	0.16	0.75	< 0.01	0.09
Foraging location	0.99	0.99	0.84	0.15	0.84	< 0.01	0.01
Foraging strategy	0.98	0.96	0.50	0.48	0.48	0.02	0.02
Roost	1.00	0.99	0.90	0.10	0.90	< 0.01	< 0.01
Size	0.98	0.95	0.91	0.07	0.88	0.03*	0.02
Skull	0.97	0.94	0.89	0.09	0.85	0.03*	0.03
Wing	0.99	0.98	0.97	0.03	0.95	0.01*	0.01
Wet season							
Diet	0.99	0.99	0.95	0.04	0.95	< 0.01	0.01
Foraging location	0.99	0.99	0.96	0.03	0.96	< 0.01	0.01
Foraging strategy	0.99	0.95	0.52	0.47	0.48	0.04	0.01
Roost	0.99	0.99	0.91	0.08	0.91	< 0.01	0.01
Size	0.93	0.90	0.75	0.18	0.72	0.03	0.07
Skull	0.94	0.91	0.77	0.17	0.74	0.03	0.06
Wing	0.97	0.96	0.88	0.09	0.87	0.01	0.03
