# Supplementary material for: Environmental and spatial drivers of taxonomic, functional, and phylogenetic characteristics of bat communities in human-modified landscapes
Source: PeerJ. 2016 Oct 13;4:e2551. doi: 10.7717/peerj.2551 (PMC5068362; doi:10.7717/peerj.2551)
Supplement: Table S8 [file peerj-04-2551-s008.pdf]

Results from variation partitioning for functional or phylogenetic composition.

Table S8. For each combination of season and scale, adjusted percentages of unique contributions of environmental [a] and spatial [c] predictors for functional or phylogenetic composition. Fraction [b] is the shared contribution of environmental and spatial predictors, and fraction [d] is the residual variation. Negative values of [b] can occur when explanatory variables are correlated but have strong and opposite effects on the response variable, or when explanatory variables have a weak correlation with the response variable but strong correlation with other explanatory variables that are correlated with the response variable (Peres-Neto et al. 2006). Testable model fractions (i.e. unique contributions) that were significant are indicated by superscript symbols (@, 0.10 ≥ P > 0.05; *, P ≤ 0.05).

	1 km scale				3 km scale				5 km scale			
	[a]	[b]	[c]	[d]	[a]	[b]	[c]	[d]	[a]	[b]	[c]	[d]
Dry season												
Functional — all	1.85	-1.09	< 0.01	0.24	2.02	-1.44	0.35	0.07	2.01	-1.09	0.01	0.07
Functional — diet	1.64	-1.43	0.23	0.56	1.90	-1.29	0.09	0.30	1.41	-1.21	0.01	0.79
Functional — foraging location	2.24	-1.47	0.08	0.16	2.20	-1.52	0.12	0.20	2.22	-1.43	0.03	0.18
Functional — foraging strategy	2.59	-2.22	0.20	0.43	2.05	-2.04	0.02	0.97	2.59	-2.34	0.32	0.44
Functional — roost	1.91	-1.74	< 0.01	0.83	2.12	-2.01	0.26	0.62	2.44	-2.34	0.60	0.30
Functional — size	2.52	-2.52	0.33	0.67	1.18	-2.56	0.38	2.00	2.50	-2.60	0.42	0.68
Functional — skull	2.34	-2.34	0.27	0.73	1.04	-2.43	0.37	2.03	2.42	-2.49	0.42	0.65
Functional — wing	2.92	-2.66	0.28	0.46	1.77	-2.53	0.15	1.61	2.80	-2.59	0.21	0.59
Phylogenetic	2.49	-2.10	0.01	0.60	2.52	-2.32	0.24	0.57	2.68	-2.37	0.28	0.41
Wet season												
Functional — all	2.28	-1.63	0.02	0.33	1.76	-2.54	0.93*	0.85	2.23	-1.76	0.15	0.38
Functional — diet	1.83	-1.30	< 0.01	0.47	1.76	-2.19	0.89@	0.55	2.00	-1.35	0.05	0.30
Functional — foraging location	2.29	-1.48	0.01	0.18	1.78	-2.12	0.64@	0.69	2.23	-1.50	0.03	0.24
Functional — foraging strategy	2.87	-2.23	0.07	0.29	2.68	-2.16	< 0.01	0.48	2.97	-2.58	0.41	0.19
Functional — roost	1.75	-2.17	< 0.01	1.41	2.58	-2.92	0.75	0.59	2.45	-2.17	0.01	0.71
Functional — size	2.68	-2.22	0.04	0.50	1.97	-2.88	0.69*	1.21	2.75	-2.52	0.33	0.44
Functional — skull	2.62	-2.11	0.03	0.46	1.96	-2.81	0.73*	1.12	2.67	-2.43	0.35	0.41
Functional — wing	2.53	-2.05	0.01	0.51	2.16	-2.62	0.59@	0.88	2.82	-2.48	0.45@	0.21
Phylogenetic	2.60	-2.02	0.01	0.42	2.27	-2.31	0.29	0.74	2.82	-2.36	0.35	0.19
