# Supplementary material for: Environmental and spatial drivers of taxonomic, functional, and phylogenetic characteristics of bat communities in human-modified landscapes
Source: PeerJ. 2016 Oct 13;4:e2551. doi: 10.7717/peerj.2551 (PMC5068362; doi:10.7717/peerj.2551)
Supplement: Table S9 [file peerj-04-2551-s009.pdf]

Spatial autocorrelation of functional or phylogenetic dispersion.

Table S9. Spatial autocorrelation of functional dispersion (based on all niche axes and individual niche axes) and phylogenetic dispersion of bat communities in the Caribbean lowlands of Costa Rica for the dry and wet seasons. Significance ($p \leq 0.05$) indicates spatial autocorrelation.

	Observed Moran's I	Expected Moran's I	SD	p
Dry season				
Functional — all	-0.02	-0.07	0.06	0.38
Functional — diet	-0.13	-0.07	0.06	0.29
Functional — foraging location	-0.03	-0.07	0.05	0.39
Functional — foraging strategy	-0.02	-0.07	0.06	0.34
Functional — roost	-0.09	-0.07	0.06	0.73
Functional — size	-0.11	-0.07	0.06	0.54
Functional — skull	-0.10	-0.07	0.06	0.56
Functional — wing	-0.09	-0.07	0.05	0.72
Phylogenetic	-0.08	-0.07	0.05	0.82
Wet season				
Functional — all	-0.08	-0.07	0.05	0.80
Functional — diet	-0.06	-0.07	0.06	0.88
Functional — foraging location	-0.05	-0.07	0.05	0.65
Functional — foraging strategy	0.00	-0.07	0.05	0.20
Functional — roost	-0.09	-0.07	0.06	0.80
Functional — size	-0.10	-0.07	0.05	0.63
Functional — skull	-0.09	-0.07	0.05	0.75
Functional — wing	-0.08	-0.07	0.05	0.92
Phylogenetic	-0.13	-0.07	0.06	0.25
