# Supplementary material for: Environmental and spatial drivers of taxonomic, functional, and phylogenetic characteristics of bat communities in human-modified landscapes
Source: PeerJ. 2016 Oct 13;4:e2551. doi: 10.7717/peerj.2551 (PMC5068362; doi:10.7717/peerj.2551)
Supplement: Table S10 [file peerj-04-2551-s010.pdf]

Spatial autocorrelation of landscape characteristics at each of three focal scales.

Table S10. Spatial autocorrelation of landscape characteristics at each of three focal scales (i.e. 1, 3, and 5 km radius) in the Caribbean lowlands of Costa Rica. Significance ($p \leq 0.05$) indicates spatial autocorrelation.

	1 km scale				3 km scale				5 km scale			
	Observed Moran's I	Expected Moran's I	SD	p	Observed Moran's I	Expected Moran's I	SD	p	Observed Moran's I	Expected Moran's I	SD	p
Percent forest	-0.04	-0.07	0.06	0.54	-0.06	-0.07	0.06	0.89	-0.05	-0.07	0.06	0.64
Percent pasture	-0.07	-0.07	0.06	0.93	-0.06	-0.07	0.06	0.78	0.02	-0.07	0.06	0.12
Mean forest patch size	-0.12	-0.07	0.05	0.37	-0.11	-0.07	0.05	0.38	-0.11	-0.07	0.06	0.50
Forest patch density	-0.09	-0.07	0.05	0.64	-0.09	-0.07	0.05	0.74	-0.08	-0.07	0.04	0.80
Simpson's diversity	-0.14	-0.07	0.05	0.24	-0.13	-0.07	0.05	0.26	-0.04	-0.07	0.05	0.56
Mean forest proximity	-0.08	-0.07	0.05	0.84	-0.05	-0.07	0.05	0.72	0.03	-0.07	0.06	0.06
Mean forest nearest neighbor	-0.04	-0.07	0.05	0.52	-0.02	-0.07	0.05	0.33	-0.03	-0.07	0.05	0.42
Mean forest patch shape	-0.09	-0.07	0.05	0.77	-0.06	-0.07	0.06	0.85	0.01	-0.07	0.05	0.15
Forest edge density	-0.05	-0.07	0.06	0.75	-0.04	-0.07	0.06	0.57	-0.02	-0.07	0.06	0.34
